# Characterising an implementation intervention in terms of behaviour change techniques and theory: the ‘Sepsis Six’ clinical care bundle

**DOI:** 10.1186/s13012-015-0300-7

**Published:** 2015-08-08

**Authors:** Siri Steinmo, Christopher Fuller, Sheldon P. Stone, Susan Michie

**Affiliations:** Department of Clinical, Educational and Health Psychology, University College London, 1-19 Torrington Place, London, UK; Department of Infection Control, University College London, London, UK; University College London Medical School, Rowland Hill Street, London, UK

## Abstract

**Background:**

Sepsis is a major cause of death from infection, with a mortality rate of 36 %. This can be halved by implementing the ‘Sepsis Six’ evidence-based care bundle within 1 h of presentation. A UK audit has shown that median implementation rates are 27–47 % and interventions to improve this have demonstrated minimal effects. In order to develop more effective implementation interventions, it is helpful to obtain detailed characterisations of current interventions and to draw on behavioural theory to identify mechanisms of change. The aim of this study was to illustrate this process by using the Behaviour Change Wheel; Behaviour Change Technique (BCT) Taxonomy; Capability, Opportunity, Motivation model of behaviour; and Theoretical Domains Framework to characterise the content and theoretical mechanisms of action of an existing intervention to implement Sepsis Six.

**Methods:**

Data came from documentary, interview and observational analyses of intervention delivery in several wards of a UK hospital. A broad description of the intervention was created using the Template for Intervention Description and Replication framework. Content was specified in terms of (i) component BCTs using the BCT Taxonomy and (ii) intervention functions using the Behaviour Change Wheel. Mechanisms of action were specified using the Capability, Opportunity, Motivation model and the Theoretical Domains Framework.

**Results:**

The intervention consisted of 19 BCTs, with eight identified using all three data sources. The BCTs were delivered via seven functions of the Behaviour Change Wheel, with four (‘education’, ‘enablement’, ‘training’ and ‘environmental restructuring’) supported by the three data sources. The most frequent mechanisms of action were reflective motivation (especially ‘beliefs about consequences’ and ‘beliefs about capabilities’) and psychological capability (especially ‘knowledge’).

**Conclusions:**

The intervention consisted of a wide range of BCTs targeting a wide range of mechanisms of action. This study demonstrates the utility of the Behaviour Change Wheel, the BCT Taxonomy and the Theoretical Domains Framework, tools recognised for providing guidance for intervention design, for characterising an existing intervention to implement evidence-based care.

**Electronic supplementary material:**

The online version of this article (doi:10.1186/s13012-015-0300-7) contains supplementary material, which is available to authorized users.

## Background

‘Sepsis’ is common and deadly and is a major cause of death from infection through shock and multiple organ failure. It has a mortality rate of 36 % [1], which rises 8 % for every 1 h delay in treatment [2], and a worldwide incidence of 300 per 100,000 population [3]. It consumes 50 % of critical care resources in the UK [4]. Mortality can be halved if sepsis is treated within the hour of presentation by implementing the ‘Six Steps of Sepsis Treatment’, known as the ‘Sepsis Six’ clinical care bundle [5]. This consists of giving high flow oxygen, administering intravenous fluids and antibiotics, and measuring blood cultures, lactate levels and urine output [2, 6]. For every five patients treated with the Sepsis Six, one life is saved [4].

Implementing the care bundle and reducing the mortality of sepsis has become a UK and international standard and priority [7–9]. The UK Sepsis Trust has estimated that each patient treated this way results in a cost saving of £3600, and a 3.4-day reduction in length of stay (including two fewer days spent in intensive care). If the Sepsis Six bundle were implemented for 80 % of septic patients in the UK, this could save 10,000 lives and release £170 million of cost savings for the National Health Service (NHS) each year [9]. However, implementation in the UK is poor, with a national audit of practice in 4500 cases (160 hospitals) reporting median implementation rates of 27–47 % for individual components of the Sepsis Six [10].

Published reports of interventions to improve implementation of the Sepsis Six show small and un-sustained effects. One programme achieved implementation levels of 39 % [6] which fell to 23 % a year later [11]. In 2010, the board of an NHS hospital in London set a target of implementing the bundle for 95 % of septic patients and made this one of its three top quality improvement areas. They created a Sepsis Board and appointed a Patient Safety Facilitator to lead implementation and deliver the intervention in three pilot areas (Accident & Emergency, Renal and Medical Assessment). Once the 95 % target was reached, it was intended to extend the implementation of this evidence-based practice to the rest of the hospital.

Over a 4-year period, this multi-component Sepsis Six implementation intervention was designed through trial and error without the use of theory. Content was not fully reported, but broadly the intervention took the form of introductory group education and training, target setting, audit, group feedback of audit results, individual personalised feedback to staff involved in incidents when the bundle was not fully implemented, and environmental changes including promotional documents and materials to aid implementation such as a ‘sepsis trolley’ or ‘sepsis bag’ containing the necessary equipment. Implementation rose from 20 % at baseline to between 80 and 90 % at the time of data collection for this study, and sepsis mortality dropped from 22 to 12 % (unpublished data c/o Royal Free Hospital Patient at Risk Team, Devaney, Stapleton, Stanley, 2013). This took 4 years and the 95 % target was not reached for all pilot areas, raising the question of how best to develop the current intervention to achieve and sustain the 95 % target before extending implementation elsewhere to the rest of the hospital.

The experience of implementing the Sepsis Six is consistent with the experience of interventions to translate research into practice in other areas which generally demonstrate limited and variable success [12, 13]. This may be in part due to a lack of explicit rationale for intervention choice [14, 15] and precise reporting of what is delivered [16], despite calls for use of theory to understand and target mechanisms of action and comprehensive reporting [17–19]. The lack of theoretical rationale and detailed reporting both hinder efforts to design, replicate or improve interventions and thus derive maximum benefit from advances in research [20, 21].

Tools have been developed by behavioural and health services research scientists to support the detailed characterisation of interventions in terms of their content, and theoretical mechanisms of action, i.e. how they produce their effects.

The Template for Intervention Description and Replication (TIDieR) is a checklist for reporting and understanding the general content of behaviour change interventions including what is delivered, to whom, by whom, when and by what mode of delivery.

**Fig. 1:**
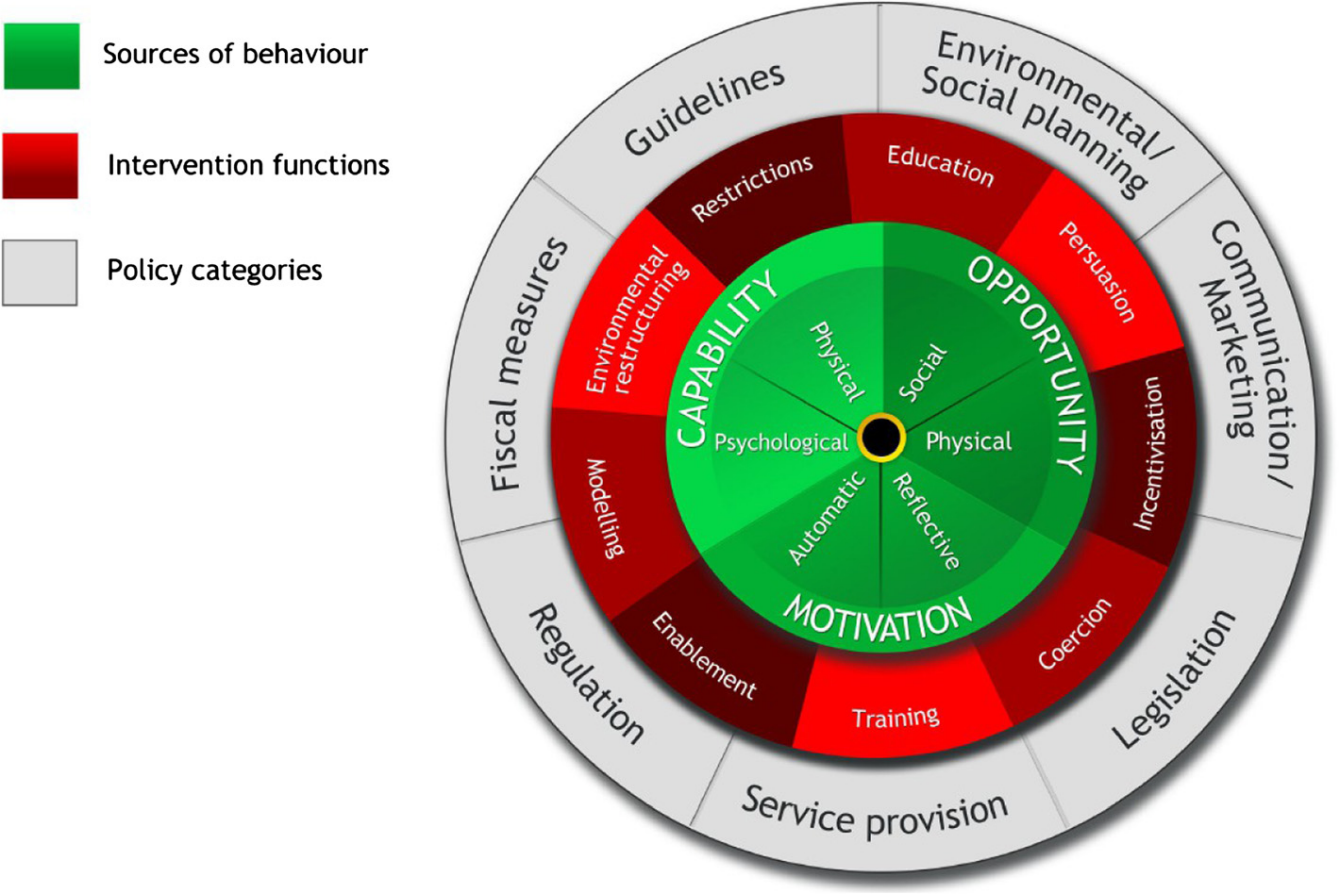
The Behaviour Change Wheel

The Behaviour Change Wheel (see Fig. 1) is a comprehensive framework for designing interventions [22] derived from an integration of 19 behaviour change frameworks. The wheel consists of three layers. At the centre, the Capability (physical and psychological), Opportunity (social and physical), Motivation (automatic and reflective) model identifies the conditions required in order for behaviour to occur and provides a method for understanding what needs to change in order for a desired behaviour to occur. This layer can be divided into a further 14 domains comprising the Theoretical Domains Framework (TDF) [23] (see Additional file 1). The TDF is derived from a synthesis of 33 theories of behaviour and was designed to make psychological theory more accessible to those working in the field of implementation. It suggests 14 key theoretical domains or determinants of behaviour change where interventions might focus; these are an elaboration of the six COM-B segments, especially the reflective motivation segment.

The second layer of the Behaviour Change Wheel comprises nine intervention ‘functions’ (see Fig. 1) which are the general means by which an intervention might change behaviour. Examples are ‘persuasion’ and ‘incentivisation’. The outer layer of the wheel identifies the policy categories that can be used to support the delivery of these functions.

The Behaviour Change Technique (BCT) Taxonomy (v1) has been developed to standardise the reporting of intervention content, that is their potentially ‘active ingredients’ [24]. It is a structured list of 93 irreducible and discrete intervention components. A BCT is the smallest component of an intervention that may have the potential to change behaviour. Each intervention function is likely to consist of several BCTs; on the other hand, any one BCT may serve several functions. For example, the BCT to give information about the health consequences of performing a behaviour may serve the function of ‘education’ and/or ‘persuasion’ depending on the context and specific message.

Health professional behaviour in a range of clinical areas has been investigated using the TDF, including diagnostic tests and imaging [25–27], prescribing errors [28], transfusions [29] and hand hygiene [30, 31]. One systematic review by Laba and colleagues [32] used both the Behaviour Change Wheel and an earlier version of the BCT Taxonomy to classify strategies used to improve adherence to cardiovascular medications across 14 studies [33]. Examples of using the BCT Taxonomy to guide intervention development on the basis of TDF analyses are seen in antibiotic prescribing [34] and organ donation [35]. A common scenario in clinical practice is that of having interventions in place which have achieved some level of success, but are not fully reported or understood and may require improvement. The usefulness of these tools for describing and modifying existing interventions has yet to be investigated.

The aims of this study, therefore, were (a) to use the Behaviour Change Wheel functions and the BCT Taxonomy (v1) to report the content of an existing hospital intervention to implement the Sepsis Six clinical care bundle and (b) to use the TDF to characterise its potential theoretical mechanisms of action. This work is a first step in designing a more effective intervention for wider implementation using these tools. It will be proceeded by a TDF-based qualitative study which will explore experiences of health care professionals receiving the intervention including barriers and levers to Sepsis Six implementation and modification of the intervention’s content based on these data.

## Methods

### Procedure and sources of data

We collected data on intervention content using three different methods:

1. *Unstructured observations*. Two researchers observed the Patient Safety Facilitator delivering 16 group education and feedback sessions with staff and one simulation training event between October 2013 and February 2014. Unstructured field notes were taken during observations that detailed what the facilitators said, questions asked by staff and how they were answered. Field notes were compared between both researchers to ensure no content was missed.
2. *Document analysis*. Both researchers read and re-read written documents describing intervention content, including the bundle protocol, promotional/educational materials and a timeline, which detailed when components were introduced. Field notes detailing the content of the documents were taken.
3. *Semi-structured interviews*. We conducted two group interviews with the Patient Safety Facilitator who delivered the intervention and two Sepsis Board nurses who helped design the intervention. Participants were asked to describe in detail their Sepsis Six improvement programme specifying exactly what was delivered, with what aim, how much, to whom, by whom and by what mode of delivery. Field notes were taken by two researchers at the time of interview and compared. Notes from interviews were collated into a narrative description of the intervention according to those who designed and delivered it. This was verified by the interview participants and amended iteratively until it was agreed that it was an accurate account of intervention content.

### Data analysis

#### Intervention content

*Step 1*. Using the TIDieR framework [21], we created a broad outline of the intervention that included the content delivered, to whom and by whom, why, by what mode of delivery and how often. Data from all three data sources were used.

*Step 2*. We extracted BCTs from the field notes (from observation and document review) and the narrative description (from interviews) using the BCT Taxonomy (v1) [24]. The resulting list of BCTs was tabulated according to whether it was identified using observation, documentary analysis or by interview.

*Step 3*. BCTs were then linked to the intervention functions of the Behaviour Change Wheel. We used guidance on the links between intervention functions and BCTs published in the *Behaviour Change Wheel Guide to Designing Interventions* [36] (see Additional file 2). Some BCTs can serve more than one function. For example, the BCT ‘adding objects to the environment’ could be linked to the ‘enablement’ and ‘environmental restructuring’ functions depending on what the object was and in what context it was delivered. In these cases, we used consensus between three of the authors (SS, CF, SM) about which functions BCTs were likely to have served guided by our knowledge of their context.

#### Mechanisms of action

*Step 4*. BCTs were mapped to TDF domains and corresponding Capability, Opportunity, Motivation conditions using (1) published expert consensus on BCTs judged ‘useful’ for influencing each TDF domain [37], (2) a more recent expert consensus exercise that has mapped 12 of the domains to 59 BCTs [38] reproduced in the *Behaviour Change Wheel Guide* [36] (see Additional file 3), and (3) consensus between the three researchers about which domains the BCTs were likely to have targeted. Two behavioural scientists (CW and LA) with expert experience with the tools, but no knowledge of the intervention, then reviewed the mapping as an extra validation. Disagreements or questions that could not easily be resolved were referred to a senior member of the study team (SM). The final results were agreed by the authors.

## Results

### Intervention content

Table 1 summarises the general content of the intervention, using the TIDieR framework. It consisted of the following broad components: group education and training, promotional and educational documents, materials provided to aid implementation, on-going group audit and feedback, and individual feedback for staff involved in incidents where the bundle was not fully implemented.

**Table 1:** Sepsis Six intervention summary based on TIDieR, delivered by Patient Safety Facilitator

Intervention components	Rationale	Mode of delivery	Delivered to	When/how often
Sepsis Six introductory education sessions including target setting of 95 % implementation	To familiarise staff with the bundle and generate enthusiasm	Face-to-face (group)	Doctors and nurses	Once when Sepsis Six is first introduced and once at each new/junior staff induction to the ward
Training (septic patient simulation) n.b. co-delivered by specialist simulation trainer and Patient Safety Facilitator	To train staff on how to implement	Face-to-face (group)	Minority of doctors and nurses (ad hoc)	Ad hoc, approximately bi-monthly
Promotional and educational documents^a^	To educate staff about the pathway and promote self-monitoring	Documents	Doctors and nurses	Ongoing
Materials provided to aid implementation^b^	To make implementation more convenient	Environment changes	Resources varied between wards	Ongoing
Audit and group feedback - daily implementation rates displayed in staff break area and verbal feedback given	To focus staff on targets and progress	Rates displayed, feedback delivered face-to-face (group)	All available doctors and nurses (majority nurses) on shift	Rates displayed daily, weekly or bi-weekly feedback sessions
Individual personalised feedback to staff involved in incidents when bundle was not fully implemented	To target specific incidents of non-compliance	Face-to-face (group)	Staff involved in incidents where bundle was not correctly or fully implemented	Ad hoc, ~2 staff per week

^a^Documents included protocol documents, checklists, Sepsis Six branding (stickers, posters, smart phone app) and sepsis folder for documentation

^b^Resources/materials include sepsis trolley (A&E) or sepsis bag (other wards) containing all necessary instruments, and antibiotic cupboard

We identified 19 BCTs and seven Behaviour Change Wheel functions in the intervention (Table 2). Eight BCTs and four functions were identified using all three data sources. All BCTs and functions in the intervention were identified using observational and interview data, with 18 of 19 BCTs and six of seven functions identified by observation alone. Interviews failed to identify three BCTs and one function. The least content was derived from documentary evidence, with no BCT or function identified using documentary evidence alone.

**Table 2:** BCTs used in the Sepsis Six intervention derived from three data sources

BCTs supported by three data sources	BCTs supported by two data sources	BCTs supported by only one data source
Information on health consequences	Demonstration of behaviour (D, I)	Focus on past success (O)
Goal setting - behaviour	Salience of consequences (O, I)	Discrepancy b/t current behaviour and goal (O)
Goal setting - outcome	Feedback - behaviour (O, I)	Verbal persuasion about capability (O)
Behavioural practice	Feedback - outcome (O, I)	
Adding objects to environment	Problem solving (O, I)	
Instruction on how to perform behaviour	Social reward (O, I)	
Prompts/cues	Social support (O, I)	
Self-monitoring behaviour	Social comparison (O, I)	
Functions supported by three data sources	Functions supported by two data sources	Functions supported by one data source
Education	Persuasion (O, I)	Incentivisation (O)
Enablement	Modelling (D, I)	
Environmental restructuring		
Training		

*O* observation, *I* interview, *D* documentary

Within the introductory group education and training component, 11 BCTs serving five functions were identified; within the documents and materials component, four BCTs serving two functions; within the group audit and feedback, seven BCTs serving four functions; and within the individual component, two BCTs serving three functions.

### Mechanisms of action

Intervention content linked to all components of Capability, Opportunity, Motivation, especially psychological capability (*n* = 15) and reflective motivation (*n* = 17), and to 13 of 14 TDF domains, suggesting that its influence on Sepsis Six implementation may have been mediated by several pathways (Table 3). The most frequent TDF domain was ‘knowledge’ (*n* = 10), which was mostly targeted via the function of ‘education’ using BCTs instruction on how to perform behaviour, information about health consequences and *feedback.* The next most frequent domains were ‘beliefs about consequences’ (*n* = 8), which was mostly targeted via the function of 'persuasion' using *BCTs information about health consequences* and *feedback on behaviour and outcome*, and ‘beliefs about capabilities’ (*n* = 8), which was mostly targeted via the functions of ‘persuasion’ and ‘enablement’ using a variety of BCTs. ‘Skills’, ‘optimism’ and ‘reinforcement’ were the most infrequently targeted theoretical domains, and the only domain not targeted in the intervention was ‘intention’.

**Table 3:** Characterising intervention content and mechanisms of action using the BCT taxonomy (v1); behaviour change wheel; capability, motivation, behaviour model; and TDF

Intervention component		Intervention content				Mechanisms of action	
		BCTs		Functions		COM-B	TDF
Group introductory education and training sessions delivered to staff in groups							
Discussion on severity and susceptibility of sepsis		Information about health consequences		Education		Psy C	Kn
Instruction on how and when to implement		Instruction on how to perform behaviour		Education		Psy C	Kn
Story of a young patient who had died from sepsis is told		Information about health consequences		Persuasion		Ref M, Auto M	B Con, Em,
Discussion of good implementation on other wards		Social comparison		Persuasion		Soc O, Ref M	SI, B Cap, S/P Id
Evidence for the efficacy of Sepsis Six for improving patient outcomes given		Information about health consequences		Education, persuasion		Psy C, Ref M	Kn, B Con
Sepsis Six simulation training (observation and practice)		Demo of behaviour, instruction on how to perform behaviour, behavioural practice, habit formation		Modelling, education, training		Phys C, Psy C	Sk, Kn, MAD, BR
Discussion on ease of implementation		Verbal persuasion about capability		Persuasion		Ref M	B Cap, Opt
Video provided instruction on six steps, patient story and praise for staff who had treated patient		Instruction on how to perform behaviour, information about health consequences, social reward		Education, persuasion, incentivisation		Psy C, Ref M, Auto M	Kn, B Con, Em, Reinf.
Ward target of SUI reduction by 50 % set		Goal setting (outcome)		Enablement		Ref M	Goal
Ward target of implementation for 95 % of patients set		Goal setting (behaviour)		Enablement		Ref M	Goal
Staff are encouraged to have legitimate authority to commence Sepsis Six using their clinical discretion (regardless of role)		Social support (unspecified)		Enablement		Soc O, Ref M	SI, S/P Id, B Cap
Staff are encouraged to seek support from superiors and facilitators regarding implementation issues		Social support (unspecified)		Enablement		Soc O	SI
Documents and materials provided to aid implementation							
Sepsis trolley and sepsis bags contained all instruments required to implement pathway		Adding objects to environment, prompts/cues		Environmental restructuring, enablement		Psy C, Phys O	MAD, Env
Cupboards contained all antibiotics likely to be needed		Adding objects to environment		Environmental restructuring		Phys O	Env
Sepsis Six logo displayed throughout wards		Adding objects to environment, prompts/cues		Environmental restructuring		Psy C, Phys O	MAD, Env
Intranet resource provided instruction on implementation		Instruction on how to perform behaviour		Education		Psy C	Kn
Antibiotics protocol provided instruction on appropriate antibiotic selection		Instruction on how to perform behaviour		Education		Psy C	Kn
Six-step checklist provided visual prompt and included checklist for completion of each step		Self-monitoring, prompts/cues		Enablement, environmental restructuring		Psy C	MAD, BR
Smartphone app provided instruction on implementation and timer for monitoring step completion		Prompts/cues, instruction on how to perform behaviour, self-monitoring		Education, enablement		Psy C	MAD, Kn, BR
Ongoing group-level audit and feedback							
Daily implementation rates displayed in staff room and updated daily		Monitoring of behaviour by others		Persuasion		Ref M	B Con, B Cap
Comparison of current performance with 95 % target made		Discrepancy b/t behaviour and goal		Enablement		Ref M	Goal
Verbal feedback implementation rates given		Feedback on behaviour		Education, persuasion		Psy C, Ref M	Kn, B Con
Generation of solutions for better implementation (staff and facilitator cooperative planning)		Problem solving		Enablement		Psy C, Soc O	BR, SI, B Cap
Reporting of patient outcomes data		Feedback on outcome of behaviour		Persuasion		Ref M	B Con
Clinical follow-up for patients who received Sepsis Six		Feedback on outcome of behaviour		Persuasion		Ref M, Auto M	B Con, Em
Discussion of past targets hit		Focus on past success		Persuasion		Ref M	B Cap
Praise from the board and facilitators for improvements made and targets reached		Social reward		Incentivisation		Ref M, Auto M	B Cap, Reinf.
Individual intervention for cases of non-implementation							
Verbal feedback on individual performance given		Feedback on behaviour		Education, persuasion		Psy C, Ref M	Kn, B Con
Generation of solutions for better individual implementation (cooperative planning with facilitator)		Problem solving		Enablement		Psy C, Soc O, Ref M	BR, SI , B Cap

TDF domain abbreviations: *Sk* skills, *Kn* knowledge, *MAD* memory, attention and decision processes, *BR* behavioural regulation, *SI* social influences, *Env* environmental context and resources, *Reinf* reinforcement, *B Cap* beliefs about capabilities, *B Con* beliefs about consequences, *S/P Id* social/professional role and identity, *Opt* optimism, *Goal* goals, *Em* emotions

COM-B abbreviations: *Phys C* physical capability, *Psy C* psychological capability, *Soc O* social opportunity, *Phys O* physical opportunity, *Ref M* reflective motivation, *Auto M* automatic motivation

## Discussion

We have illustrated a systematic, theory-based approach to specifying the content and possible mechanisms of action of an implementation intervention using behavioural science methodology and triangulation from different data sources. The intervention consisted of 19 BCTs and seven intervention functions that may have stimulated behaviour change through several mechanisms of action, especially ‘beliefs about consequences’ and ‘beliefs about capabilities’ (reflective motivation) and ‘knowledge’ (psychological capability).

There are limitations to the method used. First, since the intervention was not explicitly guided by a formal theory, we did not specify causal pathways between barriers and levers and its content. Though we used published guidance on suggested BCTs for intervention functions and the TDF domains [36–38], we were essentially ‘retrofitting’ the intervention to the frameworks rather than using them at the design stage. Using them at the design stage would involve identifying barriers and levers first and then using this analysis to select BCTs. In order to ‘retrofit’ intervention functions from BCTs, we drew on our own knowledge and understanding of the intervention and its context to link BCTs to the functions we judged them to serve and to their potential theoretical mechanisms of action. Anecdotally, this ‘retrofitting’ approach is being used by several UK Government departments to evaluate their current policies and intervention strategies in terms of identifying gaps. This retrospective approach demonstrates that these tools can be used to analyse interventions ‘post-hoc’ regardless of whether they have been used at the design stage, and may prove useful for analysing other existing interventions in need of improvements. Mapping between the tools may need further refining, perhaps for specific behaviours and contexts.

A second limitation is that intervention content and delivery are likely to vary across wards and between education and feedback sessions. For example, some wards were provided a sepsis trolley, which held all the materials needed to implement Sepsis Six while others with fewer septic patients were provided sepsis bags only, which did not include bags of fluid or antibiotics. The resource constraints of the study did not allow us to investigate this variation. In particular, we were not able to provide information about the frequency, dose or ‘weight of importance’ of each component. This complex issue of ‘amount’ as well as type of intervention requires the development of methods for measuring dimensions of intervention dose, which may also change over time [39]. The full reporting of the content in real-time, however, is a first step towards ensuring that what is intended to be delivered is in fact delivered.

A mixed method approach to data collection [36] was a strength of the study. Observation identified the largest number of BCTs and functions, and this data source revealed substantial intervention content that was not mentioned in interview. This may reflect poor recall and/or the lack of awareness people have of behaviours that have become habitual and underlines the limitations of implementation research relying only on self-reported behaviour [40, 41]. Documents revealed the least data, possibly because incomplete documentation of improvement programmes is typical of professional practice in a working hospital with competing clinical priorities and schedules. Our study clearly demonstrates the utility of direct observation of behaviour in its natural setting for collecting real-time, real-world data. Though the other two data sources did not substantially add to the data, triangulation of the three data collection methods increased our confidence in the comprehensiveness of the description.

Insufficient description of content occurs in research as well as in clinical practice, and is well recognised in the literature on behaviour change interventions [16]. The primary strength of the current research is that we were able to describe systematically this intervention using a common language and make theoretically informed inferences about how it might bring about its effects. This is a useful first step in improving the intervention and its implementation to achieve better clinical outcomes. In the next stage of the research, this understanding of the content delivered and its potential theoretical mechanisms of action will be combined with a TDF analysis of health professionals’ experience of the intervention and barriers and levers to implementation to identify gaps and enhance the existing intervention. A full description of the updated intervention using these tools will be created so that fidelity of delivery can be assessed. This approach is in keeping with the importance of providing a transparent and robust rationale for developing intervention content and for clear and full reporting.

These tools have been used across many clinical domains for diagnosing implementation problems and informing intervention development, as described in the ‘Background’. To the authors’ knowledge, this is the first report that describes a single existing intervention using the Behaviour Change Wheel, the BCT Taxonomy and the TDF, together with the recent TIDieR statement [21]. This approach is in line with calls for improved methods for applying theory to intervention design including increased understanding of how BCTs exert their influences [42, 43]. This work therefore contributes to accumulating evidence on the linking of intervention content to mechanisms of action [36–38, 44]. Further research should evaluate the usefulness of these tools for ‘retrospective’ analyses of different interventions targeting different behaviours in different settings.

## Conclusion

This study demonstrates how the use of a variety of information sources, particularly observation, and tools developed to make behavioural theory and implementation accessible to non-specialists can be used to specify the content and possible mechanisms of action of existing behaviour change interventions which, although designed without the use of theory, have achieved some level of success in clinical practice but require improvement. This enables comprehensive thinking about current practice and the drivers of behaviour and thus provides a sound platform for intervention improvement and replication.

## Additional files

Additional file 1:
**Figure- TDF domains linked to Capability, Opportunity, Motivation components.** Figure depicting the central hub of the Behaviour Change Wheel (the Capability, Opportunity, Motivation Model) and how these components can be further divided into TDF domains (PDF 254 kb) Additional file 2:
**Matrix of relevant BCTs for intervention functions.** Table outlining frequently used BCTS for each intervention function. Used for data analysis linking BCTs identified back to intervention functions. From Michie et al. [36] (PDF 62 kb) Additional file 3:
**Expert consensus linking BCTs to TDF domains.** Table outlining how BCTs link to TDF domains, i.e. which BCTs are recommended for use for targeting each of the TDF domains. Used for data analysis linking BCTs identified to their potential TDF mechanisms of action (PDF 54 kb)

## Abbreviations

BCT: Behaviour Change Technique
NHS: National Health Service
TDF: Theoretical Domains Framework
TIDieR: Template for Intervention Description and Replication
