# Supplementary figures and images for: Characterising an implementation intervention in terms of behaviour change techniques and theory: the ‘Sepsis Six’ clinical care bundle
Source: Implement Sci. 2015 Aug 8;10:111. doi: 10.1186/s13012-015-0300-7 (PMC4529730; doi:10.1186/s13012-015-0300-7)

Additional File 1

Figure- TDF domains linked to Capability, Opportunity, Motivation components

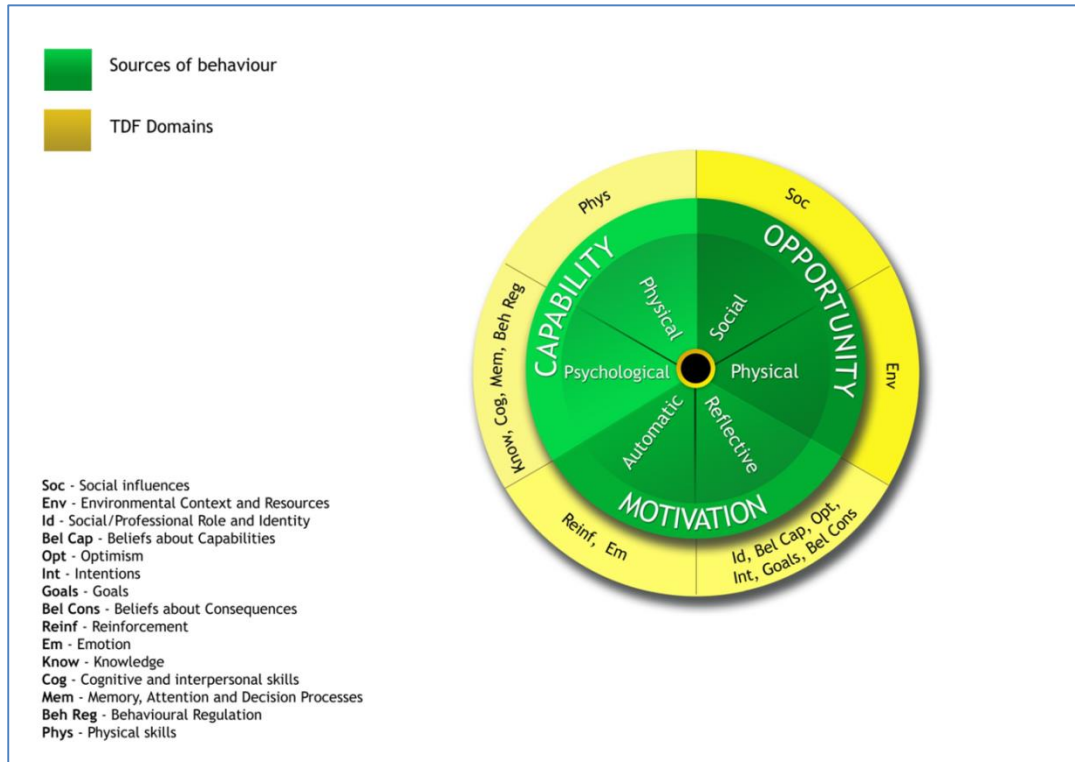

Supplement: Additional file 1: — Figure- TDF domains linked to Capability, Opportunity, Motivation components. Figure depicting the central hub of the Behaviour Change Wheel (the Capability, Opportunity, Motivation Model) and how these components can be further divided into TDF domains (PDF 254 kb) [file 13012_2015_300_MOESM1_ESM.pdf]
