# Supplementary material for: Characterising an implementation intervention in terms of behaviour change techniques and theory: the ‘Sepsis Six’ clinical care bundle
Source: Implement Sci. 2015 Aug 8;10:111. doi: 10.1186/s13012-015-0300-7 (PMC4529730; doi:10.1186/s13012-015-0300-7)
Supplement: Additional file 2: — Matrix of relevant BCTs for intervention functions. Table outlining frequently used BCTS for each intervention function. Used for data analysis linking BCTs identified back to intervention functions. From Michie et al. [36] (PDF 62 kb) [file 13012_2015_300_MOESM2_ESM.pdf]

MS 1132100059162845

Additional File 2

Matrix of relevant BCTs for intervention functions
from Michie et al. (2014). *The Behaviour Change Wheel: A Guide to Designing Interventions.* London, Silverback.

**Intervention function**	**Individual BCTs**
**Education**	**Most frequently used BCTs:**<br>**Information about social and environmental consequences**<br>**Information about health consequences**<br>**Feedback on behaviour**<br>**Feedback on outcome(s) of the behaviour**<br>**Prompts/cues**<br>**Self-monitoring of behaviour**<br><br>Less frequently used BCTs:<br>Biofeedback<br>Self-monitoring of outcome(s) of behaviour<br>Cue signalling reward<br>Satiation<br>Information about antecedents<br>Re-attribution<br>Behavioural experiments<br>Information about emotional consequences<br>Information about others' approval

**Intervention function**	**Individual BCTs**
**Persuasion**	**Most frequently used BCTs:**<br>**Credible source**<br>**Information about social and environmental consequences**<br>**Information about health consequences**<br>**Feedback on behaviour**<br>**Feedback on outcome(s) of the behaviour**<br><br>Less frequently used BCTs:<br>Biofeedback<br>Re-attribution<br>Focus on past success<br>Verbal persuasion about capability<br>Framing/reframing<br>Identity associated with changed behaviour<br>Identification of self as role model<br>Information about emotional consequences<br>Salience of consequences<br>Information about others' approval<br>Social comparison
**Incentivisation**	**Most frequently used BCTs:**<br>**Feedback on behaviour**<br>**Feedback on outcome(s) of behaviour**<br>**Monitoring of behaviour by others without evidence of feedback**<br>**Monitoring outcome of behaviour by others without evidence of feedback**

Intervention function	Individual BCTs
	**Self-monitoring of behaviour**<br><br>Less frequently used BCTs:<br>Paradoxical instructions<br>Biofeedback<br>Self-monitoring of outcome(s) of behaviour<br>Cue signalling reward<br>Remove aversive stimulus<br>Reward approximation<br>Rewarding completion<br>Situation-specify reward<br>Reward incompatible behaviour<br>Reduce reward frequency<br>Reward alternate behaviour<br>Remove punishment<br>Social reward<br>Material reward<br>Material reward (outcome)<br>Self-reward<br>Non-specific reward<br>Incentive<br>Behavioural contract<br>Commitment<br>Discrepancy between current behaviour and goal<br>Imaginary reward

Intervention function	Individual BCTs
**Coercion**	**Most frequently used BCTs:**<br>**Feedback on behaviour**<br>**Feedback on outcome(s) of behaviour**<br>**Monitoring of behaviour by others without evidence of feedback**<br>**Monitoring outcome of behaviour by others without evidence of feedback**<br>**Self-monitoring of behaviour**<br><br>Less frequently used BCTs:<br>Biofeedback<br>Self-monitoring of outcome(s) of behaviour<br>Remove access to the reward<br>Punishment<br>Behaviour cost<br>Remove reward<br>Future punishment<br>Behavioural contract<br>Commitment<br>Discrepancy between current behaviour and goal<br>Incompatible beliefs<br>Anticipated regret<br>Imaginary punishment
**Training**	**Most frequently used BCTs:**<br>**Demonstration of the behaviour**

**Intervention function**	**Individual BCTs**
	**Instruction on how to perform a behaviour**<br>**Feedback on the behaviour**<br>**Feedback on outcome(s) of behaviour**<br>**Self-monitoring of behaviour**<br>**Behavioural practice/rehearsal**<br><br>Less frequently used BCTs:<br>Biofeedback<br>Self-monitoring of outcome(s) of behaviour<br>Habit formation<br>Habit reversal<br>Graded tasks<br>Behavioural experiments<br>Mental rehearsal of successful performance<br>Self-talk<br>Self-reward
**Restriction**	*No BCTs in BCTTv1 are linked to this intervention function because they are focused on changing the way that people think, feel and react rather than the way the external environment limits their behaviour.*
**Environmental restructuring**	**Most frequently used BCTs:**<br>**Adding objects to the environment**<br>**Prompts/cues**<br>**Restructuring the physical environment**

**Intervention function**	**Individual BCTs**
	Less frequently used BCTs:<br>Cue signalling reward<br>Remove access to the reward<br>Remove aversive stimulus<br>Satiation<br>Exposure<br>Associative learning<br>Reduce prompt/cue<br>Restructuring the social environment
**Modelling**	**Most frequently used BCTs:**<br>**Demonstration of the behaviour**
**Enablement**	**Most frequently used BCTs:**<br>**Social support (unspecified)**<br>**Social support (practical)**<br>**Goal setting (behaviour)**<br>**Goal setting (outcome)**<br>**Adding objects to the environment**<br>**Problem solving**<br>**Action planning**<br>**Self-monitoring of behaviour**<br>**Restructuring the physical environment**

Intervention function	Individual BCTs
	**Review behaviour goal(s)**<br><br>**Review outcome goal(s)**<br><br><br>Less frequently used BCTs:<br><br>Social support (emotional)<br><br>Reduce negative emotions<br><br>Conserve mental resources<br><br>Pharmacological support<br><br>Self-monitoring of outcome(s) of behaviour<br><br>Behaviour substitution<br><br>Overcorrection<br><br>Generalisation of a target behaviour<br><br>Graded tasks<br><br>Avoidance/reducing exposure to cues for the behaviour<br><br>Restructuring the social environment<br><br>Distraction<br><br>Body changes<br><br>Behavioural experiments<br><br>Mental rehearsal of successful performance<br><br>Focus on past success<br><br>Self-talk<br><br>Verbal persuasion about capability<br><br>Self-reward<br><br>Behavioural contract<br><br>Commitment<br><br>Discrepancy between current behaviour and goal

**Intervention function**	**Individual BCTs**
	Pros and cons<br>Comparative imagining of future outcomes<br>Valued self-identity<br>Framing/reframing<br>Incompatible beliefs<br>Identity associated with changed behaviour<br>Identification of self as role model<br>Salience of consequences<br>Monitoring of emotional consequences<br>Anticipated regret<br>Imaginary punishment<br>Imaginary reward<br>Vicarious consequences
