# Supplementary material for: Characterising an implementation intervention in terms of behaviour change techniques and theory: the ‘Sepsis Six’ clinical care bundle
Source: Implement Sci. 2015 Aug 8;10:111. doi: 10.1186/s13012-015-0300-7 (PMC4529730; doi:10.1186/s13012-015-0300-7)
Supplement: Additional file 3: — Expert consensus linking BCTs to TDF domains. Table outlining how BCTs link to TDF domains, i.e. which BCTs are recommended for use for targeting each of the TDF domains. Used for data analysis linking BCTs identified to their potential TDF mechanisms of action (PDF 54 kb) [file 13012_2015_300_MOESM3_ESM.pdf]

MS 1132100059162845

Additional File 3

Expert consensus linking BCTs to TDF Domains

from Cane et al. (2015). From 'Lists of behaviour change techniques (BCTs) to structured hierarchies: Comparison of two methods of developing a hierarchy of BCTs'. *British Journal of Health Psychology* **20**(1): 130-150.

TDF domain	BCT
**Knowledge**	Health consequences
	Biofeedback
	Antecedents
	Feedback on behaviour
**Skills**	Graded tasks
	Behavioural rehearsal / practice
	Habit reversal
	Body changes
	Habit formation
**Professional Role and Identity**	*No BCTs are linked to this domain*
**Beliefs about Capabilities**	Verbal persuasion to boost self-efficacy
	Focus on past Success
**Optimism**	Verbal persuasion to boost self-efficacy
**Beliefs about Consequences**	Emotional consequences
	Salience of consequences
	Covert sensitisation
	Anticipated regret
	Social and environmental consequences
	Comparative imagining of future outcomes
	Vicarious reinforcement
	Threat
	Pros and cons
	Covert conditioning
**Reinforcement**	Threat
	Self-reward
	Differential reinforcement
	Incentive
	Thinning
	Negative reinforcement
	Shaping
	Counter conditioning

TDF domain	BCT
	Discrimination training
	Material reward
	Social reward
	Non-specific reward
	Response cost
	Anticipation of future rewards or removal of punishment
	Punishment
	Extinction
	Classical conditioning
**Intentions**	Commitment
	Behavioural contract
**Goals**	Goal setting (outcome)
	Goal setting (behaviour)
	Review of outcome goal(s)
	Review behaviour goals
	Action planning (including implementation intentions)
**Memory, Attention and Decision Processes**	*No BCTs are linked to this domain*
**Environmental Context and Resources**	Restructuring the physical environment
	Discriminative (learned) cue
	Prompts / cues
	Restructuring the social environment
	Avoidance / changing exposure to cues for the behaviour
**Social Influences**	Social comparison
	Social support or encouragement (general)
	Information about others' approval
	Social support (emotional)
	Social support (practical)
	Vicarious reinforcement
	Restructuring the social environment
	Modelling or demonstrating the behaviour
	Identification of self as role model
	Social reward
**Emotion**	Reduce negative emotions
	Emotional consequences
	Self-assessment of affective consequences
	Social support (emotional)
**Behavioural Regulation**	Self-monitoring of behaviour
